# Ethnicity and OPRM variant independently predict pain perception and patient-controlled analgesia usage for post-operative pain

**DOI:** 10.1186/1744-8069-5-32

**Published:** 2009-06-23

**Authors:** Ene-choo Tan, Eileen CP Lim, Yik-ying Teo, Yvonne Lim, Hai-yang Law, Alex T Sia

**Affiliations:** 1 KK Research Centre, KK Women's and Children's Hospital, Singapore; 2 Department of Psychological Medicine, Yong Loo Lin School of Medicine, National University of Singapore, Singapore; 3 Wellcome Trust Centre for Human Genetics, University of Oxford, Oxford, UK; 4 Department of Women's Anesthesia, KK Women's and Children's Hospital, Singapore; 5 Genetics Service, KK Women's and Children's Hospital, Singapore

## Abstract

**Background:**

Morphine consumption can vary widely between individuals even for identical surgical procedures. As mu-opioid receptor (OPRM1) is known to modulate pain perception and mediate the analgesic effects of opioid compounds in the central nervous system, we examined the influence of two OPRM polymorphisms on acute post-operative pain and morphine usage in women undergoing elective caesarean delivery.

**Results:**

Data on self-reported pain scores and amount of total morphine use according to patient-controlled analgesia were collected from 994 women from the three main ethnic groups in Singapore. We found statistically significant association of the OPRM 118A>G with self-administered morphine during the first 24-hour postoperative period both in terms of total morphine (p = 1.7 × 10^-5^) and weight-adjusted morphine (p = 6.6 × 10^-5^). There was also significant association of this OPRM variant and time-averaged self-rated pain scores (p = 0.024). OPRM 118G homozygotes used more morphine and reported higher pain scores than 118A carriers. Other factors which influenced pain score and morphine usage include ethnicity, age and paying class.

**Conclusion:**

Our results suggest that ethnicity and OPRM 118A>G genotype are independent and significant contributors to variation in pain perception and postoperative morphine use in patients undergoing cesarean delivery.

## Background

Studies have consistently demonstrated the existence of individual differences for both clinical and experimental pain [1-3]. These include heat-pain sensitivity, responses to thermal stimuli [4], pressure pain [5], nociceptive stimuli [6], and pain related to chronic pain conditions [7]. This interindividual variability is partly heritable, as reported in studies with twin pairs and animal models [8].

In addition to pain sensitivity and tolerance, clinical studies indicate that there is extensive interindividual variability in drug response and voluntary use of analgesics [9,10]. For opioids which are commonly used for both acute pain and chronic pain, there is wide range in interindividual requirement in the use of morphine as demonstrated by the data from patient-controlled analgesia (PCA) [11,12]. As patients are in control of the dosage delivery, the quantity of self-administered analgesics will be indicative of pain perception and tolerance.

We have previously found an association of the OPRM 118A>G SNP with pain perception and the amount of morphine self-administered in Chinese patients undergoing the same surgical procedure [13]. In another study, we found that ethnic Indian patients reported higher pain and used more morphine compared to Chinese or Malays [14]. We also previously reported that the frequency of OPRM 118G allele was higher in Indian compared to other Asian populations [15]. In the current study, we investigated the association of two polymorphisms in the OPRM gene with self-rated postoperative pain and the amount of self-administered morphine in three main Asian ethnic groups. In addition, we tested the hypothesis that OPRM variants and ethnicity are independent predictors of post-operative pain and analgesic consumption.

## Methods

### Study subjects

This study involved a prospective cohort of women undergoing elective caesarian section under spinal anesthesia at KK Women's and Children's Hospital. All study procedures were approved by the hospital institutional review board. Subjects were recruited consecutively over 28 months from May 2005 to August 2007. Written informed consent was obtained from the patients after the study was explained to them before the scheduled caesarean procedure.

### Study procedure

Data were collected on ethnicity, age, weight, height, duration of operation, and post-operative vital signs. Subjects were asked to state their ethnicity and that of their parents and all four grandparents. Information on previous lower segment cesarean section (LSCS), age, height and weight was obtained from case records. Before initiation of anesthesia, baseline blood pressure, heart rate, respiratory rate, and oxygen saturation by pulse oximetry were recorded. Intravenous access was then established with all parturients given 0.5 liter lactated Ringer's solution for prehydration. Spinal anesthesia was then induced with a standard single shot at L3-L4 or L4-L5 with the parturients in the lateral or sitting position. A 27-gauge pencil point needle was passed into the subarachnoid space and free flow of cerebral spinal fluid was ensured before the intrathecal dose of 2 ml of heavy bupivacaine 0.5% (Marcain, AstraZeneca, Sodertalje, Sweden) and 100 μg of morphine was injected. Surgery was allowed to begin when sensory block to cold at patients' midline was detected at T4 level with the patients lying in the supine position with a 15° left lateral tilt.

Only one patient had had "technical failure" due to unsuccessful attempts to obtain cerebral spinal fluid with the spinal needle. Patients who did not obtain sensory level of >T6 twenty minutes after the administration of spinal anesthesia were deemed to have a 'failed block'. Six were converted to general anesthesia due to such "failed block". All seven were withdrawn from the study. Antiemetics (IV 4 mg ondansetron, 10 mg metoclopramide, and 4 mg dexamethasone) were administered at the end of the surgery.

### Morphine consumption and side effects

On arrival at the post-anesthesia area, all patients received an IV patient controlled analgesia pump (PCA) (Rhythmic, Mircel, Greece). The PCA pump was set to deliver one mg IV bolus of morphine per demand with a lockout time of five minutes, without continuous background infusion. The maximum amount of morphine allowed was 10 mg/hour. The cumulative dose of morphine administered by each patient within every four-hour period was recorded until 24 hours post-operation. No other supplemental analgesics were given during the first 24 hours postoperative period.

Seven patients who had PCA removed before 24 hours when the last three recordings were not zero, and one patient who misinterpreted the use of PCA pump were removed from the study. Another seven patients requested for withdrawal from the study because they did not want to continue with PCA.

Other data recorded were nausea and pruritis on a severity scale of 0–3, 0 = none, 1 = mild, 2 = moderate, 3 = severe; vomiting as defined by the number of episodes; and respiratory depression as defined by a rate of < 8 and/or shallow breathing.

### Pain measures

At the postoperative recovery area and at four hourly intervals up to 24 hours counting from the time of administration of spinal anesthesia, subjects were asked to rate the degree of pain on the Visual Analogue Scale (VAS) consisting of numeric scale from zero to 10 points; with zero being "no pain" and 10 being "maximum pain".

Patients who received nitrous oxide by inhalation and/or IV fentanyl of up to 100 μg were excluded from the study, as the use of intraoperative opioids and hypnotics could potentially affect pain scores and the use of PCA morphine postoperatively. Patients who were inadvertently prescribed and took any oral analgesic were also excluded from the analysis.

### Genetic Analysis

At the time of establishing the mandatory intravenous access before surgery, three ml of blood was collected in EDTA tubes and stored. DNA was extracted in batches from frozen whole blood samples using the Gentra Puregene Blood Kit (Gentra Systems Inc., Minneapolis, USA). DNA was checked for quantity and purity using the NanoDrop Spectrophotometer (NanoDrop Technologies, Wilmington, USA).

The OPRM A118G polymorphism (rs1799971) was genotyped by the Taqman SNP Genotyping Assay ID C___8950074_1 (Applied Biosystems, Foster City, USA). Amplification was performed in a volume of 12 μl containing 25 ng genomic DNA, Taqman Universal Polymerase Chain Reaction Master Mix, 60 nM of each probe, and 270 nM of each primer. Cycling and hybridization conditions were set according to manufacturer's instructions. The 50 cycles of denaturation and annealing/extension and post-polymerase chain reaction quantification of fluorescent intensity were performed using the Applied Biosystems 7300 Real-Time PCR System.

The OPRM -172T>G promoter polymorphism (rs6912029) was genotyped by allele-specific PCR using primers CACAGAAGAGTGCCCAGTGA and GAGATACGCCAAGGCATCAGT followed by restriction with BSe8I at 60°C for hours. Following agarose gel electrophoresis, two bands (234 bp and 200 bp) would be observed for the T allele while the amplicon with the G allele would be unrestricted and remained as 434 bp.

### Statistical Analysis

Summary statistics stratified by the genotypes of the OPRM118 locus were calculated for all variables. Normality for each variable was assessed using a Shapiro-Wilk test. Each variable with distribution that did not deviate from normality was summarized using the mean and standard deviation, and comparison across the three genotypes was performed with an analysis of variance (ANOVA). For variables with skewed distributions, the median and semi-interquartile range was calculated and comparison across the three genotypes using the Kruskal-Wallis rank sum test.

Statistical significance for assessing Hardy-Weinberg equilibrium for the two OPRM polymorphisms was obtained using a Monte-Carlo permutation strategy. Multiple linear regression analysis correcting for race, age, BMI and average VAS pain score was performed to investigate the effects of OPRM polymorphisms on morphine intake per kilogram bodyweight. The amount of variance explained by each covariate was calculated by assessing the sums-of-squares decomposition in the regression. A separate multiple linear regression analysis was performed for each of the three ethnic groups, similarly correcting for age, BMI and average VAS pain score. Haplotypes for each sample at the two OPRM polymorphisms were statistically inferred using an Expectation-Maximization procedure, and haplotypic effects for the two OPRM polymorphisms were assessed. All analyses were performed using the statistical software R, and statistical significance was set at p < 0.05.

## Results

### Descriptive statistics

A total of 1066 subjects were recruited. The procedure for some subjects did not meet study requirement and their data were removed. Among them there were 24 Chinese, 12 Malays, 11 Indians, 25 mixed ancestry or of races other than Chinese, Malays and Indians. Results from 994 subjects were included in the analysis. There were 620 Chinese (62.1%), 241 Malays (24.1%) and 137 Indians (13.7%). The mean age was 32.5 years (SD = 4.8), mean weight was 70.9 kg (SD = 11.1) and mean height 1.58 m (SD = 0.06). Six-hundred and ten were private or paying patients who paid for all surgical and hospitalization expenses fully while 384 were "non-private" patients who received co-payment from the government. For 33.6% of the patients it was the first caesarean delivery. Just over 50% had a prior caesarean delivery, 14.9% had two and 0.9% had three prior caesarean deliveries. Average duration of operation was 53.7 min (SD = 16.0). There was statistically significant difference between paying (47.9 ± 13.6 min) and subsidized class (63.1 ± 15.0 min).

The distribution of the genotype and allele frequencies for the A118G polymorphism (rs1799971) was significantly different between Chinese and Malays (genotypic: p = 1.0 × 10^-7^); allelic: p = 1.2 × 10^-7^, between Chinese and Indians (genotypic: p = 9.8 × 10^-3^; allelic: p = 2.0 × 10^-3^), but not between Malays and Indians (genotypic: p = 0.374; allelic: p = 0.201).

### Morphine usage and pain measures

The average amount of morphine the patients administered on themselves through the PCA pump was 8.95 mg (SD = 9.59). Sixty-five patients did not use any, 129 patients used only one dose, while another 122 administered 2 doses. The highest amount recorded was 62 mg, followed by 55 mg. The rest were between three and 40 mg.

Average VAS score was 0.594 (SD = 0.591). Two hundred and thirty-eight patients (23.8%) selected VAS score of zero for all six four-hourly time points indicating no pain at all during the first 24 hour post-operative period. The highest time-averaged VAS over the 24-hour period was 4.

### Univariate analysis

In a univariate analysis for maximum VAS scores, there was statistically significant association with both race (p = 4.2 × 10^-5^) and age (p < 10^-3^), with Indians and younger patients reporting higher maximum pain scores. For mean VAS scores, race (p = 2.1 × 10^-7^) and age (p = 0.046) remain significant factors. Paying class was marginally significant (p = 0.048) with private patients having lower mean scores.

For weight-adjusted morphine usage, univariate analysis revealed that maximum VAS scores had the strongest association (p < 10^-16^), followed by ethnicity (p = 2.5 × 10^-14^) with those of Indian ancestry using the most morphine. Other statistically significant factors were paying class (p = 3.7 × 10^-6^) and age (p = 5.5 × 10^-4^), with private patients and those older in age using less morphine.

### Association of genetic variants with pain scores and morphine use

For VAS scores, there was statistically significant association with 118 A>G polymorphism for the time-averaged VAS scores (p = 0.024) but not across all time-points (p-values between 0.010 and 0.522) (Table 1). There was also highly significant difference among the three different genotype groups in terms of total morphine self-administered (p = 1.7 × 10^-5^) and weight-adjusted morphine (p = 6.6 × 10^-5^) (Table 1). After correcting for age, VAS scores and BMI, the association with morphine was still highly significant (p = 7.85 × 10^-3^). There was also statistically significant difference at every time-point (p-values between 0.039 and 6.8 × 10^-7^).

**Table 1:** Association of OPRM 118A>G with different variables

**Variable**	**OPRM 118 genotypes**			**p value**
	**AA**	**AG**	**GG**	
Age (years)	32.4 (4.7)	32.0 (4.8)	32.7 (5.4)	0.19
Height (cm)	157.7 (5.6)	157.5 (5.8)	157.6 (5.4)	0.88
Weight (kg)	70.2 (10.5)	70.9 (11.3)	72.4 (11.6)	0.11
BMI (kg/m^2^)	28.2 (4.1)	28.6 (4.4)	29.2 (4.5)	6.0 × 10^-5^
Duration of operation (min)	53.3 (16.3)	54.1 (15.9)	54.0 (15.0)	0.78
Morphine* (total)	4.0 (3.5)	6.0 (5.5)	7.0 (7.0)	1.7 × 10^-5^
100 × Morphine/kg bodyweight *	5.8 (5.7)	8.8 (8.2)	10.9 (10.3)	6.6 × 10^-5^
VAS score* (time averaged)	0.29 (0.29)	0.43 (0.36)	0.43 (0.36)	2.5 × 10^-2^
VAS score* (average of maximum)	1.0 (0.5)	2.0 (0.5)	2.0 (1.0)	0.19

*variables where the median and semi-interquartile range were presented instead of the mean with the standard deviation.

For the promoter polymorphism, there was no statistically significant association with mean and maximum pain scores or the score at any time-point. There was marginal association with weight-adjusted morphine used but it did not reach significance (p = 0.076). Haplotypes consisting of the two polymorphisms were also assessed although this did not improve the association with the response variable significantly.

**Figure 1:**
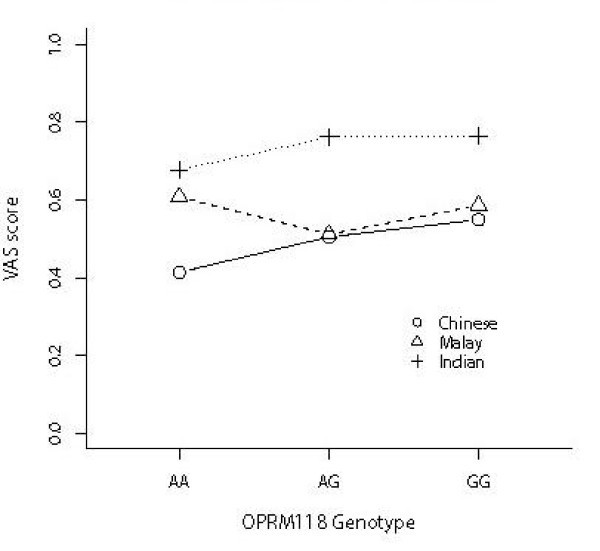
**VAS score and OPRM 118 genotypes for each of the three ethnic groups**.

**Figure 2:**
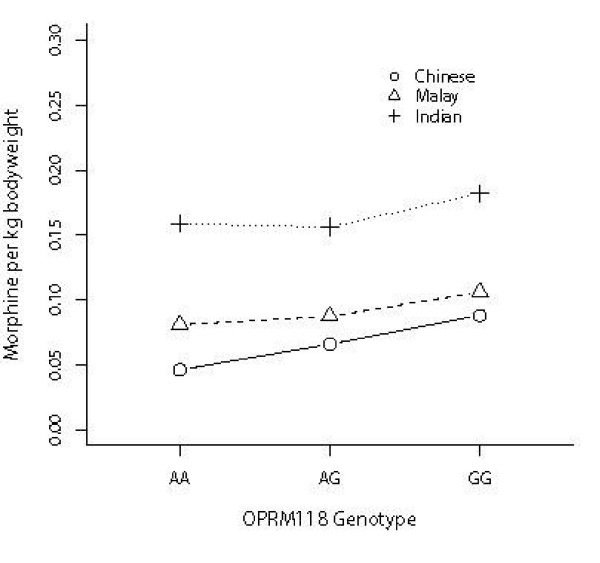
**Mean amount of morphine used (corrected for bodyweight) and OPRM 118 genotypes for each of the three ethnic groups**.

To avoid false association due to allele frequency differences among different ethnic populations, separate analysis was performed for each of the three different groups. There was no statistically significant association with the promoter polymorphism for pain scores or morphine usage in all three groups (Table 2), but for the 118A>G polymorphism statistically significant association remained for Chinese (p = 3.90 × 10^-3^) but not Malays (p = 0.085) and Indians (p = 0.804) for morphine usage (Fig 1 and Table 3). For Chinese, there was evidence suggesting that every additional copy of the G allele results in an average increase in morphine usage by 0.025 mg (95% CI: 0.012 – 0.038, p = 1.7 × 10^-4^). The trend was observed in both Malay and Indian groups (Fig 2), with corresponding increase of 0.014 mg (95% CI: -0.009 – 0.037) and 0.006 mg (95% CI: -0.032 – 0.044), although these results did not reach statistical significance.

**Table 2:** VAS and morphine use for the three OPRM -172T>G genotypic groups

VAS (average)*	**Chinese (n = 610)**	**Malays (n = 238)**	**Indians (n = 135)**	**All (n = 983)**
TT (n = 8)	0.14 (0.11)	-	1.43 (0.00)	0.14 (0.39)
GT (n = 156)	0.43 (0.43)	0.29 (0.29)	0.57 (0.38)	0.43 (0.36)
GG (n = 819)	0.29 (0.36)	0.43 (0.36)	0.71 (0.43)	0.43 (0.36)
				
Morphine/kg bodyweight (× 100)*				
TT (n = 8)	11.6 (12.6)	-	29.4 (11.5)	11.6 (15.4)
GT (n = 156)	5.3 (4.9)	8.1 (5.9)	18.9 (10.2)	6.3 (6.5)
GG (n = 819)	6.3 (6.7)	9.1 (8.5)	15.7 (11.2)	7.8 (8.0)

*Median and semi-interquartile range

**Table 3:** VAS and morphine use for the three OPRM A118A>G genotypic groups

VAS (average)*	**Chinese (n = 617)**	**Malays (n = 241)**	**Indians (n = 136)**	**All (n = 994)**
AA (n = 389)	0.29 (0.29)	0.43 (0.36)	0.71 (0.36)	0.29 (0.29)
AG (n = 435)	0.43 (0.43)	0.36 (0.29)	0.71 (0.43)	0.43 (0.36)
GG (n = 170	0.43 (0.36)	0.43 (0.29)	0.71 (0.50)	0.43 (0.36)
				
Morphine/kg bodyweight (× 100)*				
AA (n = 389)	4.6 (4.4)	8.1 (6.6)	15.9 (11.7)	5.8 (5.7)
AG (n = 435)	6.6 (7.3)	8.7 (7.8)	15.6 (11.2)	8.8 (8.2)
GG (n = 170)	8.8 (9.3)	10.6 (10.4)	18.2 (15.5)	10.9 (10.3)

*Median and semi-interquartile range

### Additional analyses

For OPRM 118 A>G, there was statistically significant association between genotypes and nausea (p = 0.026) for all patients (Table 4). This 118G allele was associated with a reduced risk of nausea in spite of a higher morphine usage. The AA group had the highest nausea score of 0.033 (SD = 0.006) while the GG group with the lowest at 0.009 (SD = 0.004). The mean for the heterozygous group was 0.029 (SD = 0.006). There was also statistically significant association of vomiting episodes with this SNP (p = 0.022), again with the AA group having the highest mean number of episodes at 0.166 (SD = 0.035) while the GG group with the lowest at 0.117 (SD = 0.042). The mean for the heterozygous group was 0.117 (SD = 0.042). However, the association with both variables was not statistically significant when patients were stratified by ethnicity with smaller numbers of subjects in each group. There was no significant association with pruritis for all patients or individual ethnic groups.

**Table 4:** Association of OPRM A118A>G with nausea and vomiting episodes

**Mean nausea score (SD)**	**Chinese (n = 617)**	**Malays (n = 241)**	**Indians (n = 136)**	**All (n = 994)**
AA (n = 389)	0.025 (0.096)	0.043 (0.141)	0.062 (0.196)	0.033 (0.006)
AG (n = 435)	0.015 (0.069)	0.044 (0.170)	0.050 (0.156)	0.029 (0.006)
GG (n = 170)	0.012 (0.067)	0.003 (0.022)	0.012 (0.063)	0.009 (0.004)
				
**No. of vomiting episodes (SD)**				
AA (n = 389)	0.160 (0.702)	0.060 (0.307)	0.360 (0.892)	0.166 (0.035)
AG (n = 435)	0.070 (0.358)	0.150 (0.560)	0.070 (2.004)	0.117 (0.042)
GG (n = 170)	0.060 (0.357)	0 (0)	0.260 (0.378)	0.041 (0.023)

For OPRM -172 T>G, there was no statistically significant association between genotypes and nausea, number of vomiting episodes or pruritis for all patients or within any ethnic subgroup.

In a multiple regression analysis, the most important factor contributing to bodyweight-adjusted morphine usage was maximum pain score (p < 10^-16^), followed by ethnicity (p = 3.1 × 10^-15^) and OPRM 118 A>G (p = 5.2 × 10^-5^). If the p-value for the association with weight-adjusted morphine was corrected for OPRM 118 A>G, ethnicity is still a significant contributing factor (p = 1.1 × 10^-10^).

## Discussion

This study examined the effect of two polymorphisms within the OPRM gene on pain perception and morphine usage. The results showed that the 118G variant was associated with higher pain scores, higher morphine usage, and lower nausea score.

Other studies on pain sensitivity have also found that carriers of 118G are more sensitive to electrical stimuli and chemically induced pain [16], and also pressure pain [17]. In terms of analgesic requirement, previous studies have shown that 118G carriers require higher amount of morphine for cancer pain (n = 99) [18], total knee arthroplasty (n = 147) [19], total hysterectomy (n = 80) [20], and major abdominal surgery [21]. Another study on cancer patients also found that those carrying at least one copy of 118G were poorer responders to morphine [22]. Compared to these studies, our study sample is the most homogeneous in that it involved patients within a narrower age range who were undergoing the same surgery; it also had the largest number of subjects. The results showed the same pattern of higher pain score and morphine usage for 118G carriers among non-laboring women undergoing caesarean section.

However, one study on labor pain showed that women carrying the 118G allele of OPRM were more sensitive to analgesic effect of intrathecal fentanyl [23]. Although this group is similar to the subjects in our study in terms of hormones related to pregnancy, the difference is they were also in labor whereas our patients were not. The authors of the study suggest that the increased analgesic effect of the 118G allele could be due to the increased binding in response to intrathecal fentanyl, or that the mechanism of spinal and systemic opioid pharmacokinetics may be different; even though the latter was not supported by another study involving patients who had laparoscopic abdominal surgery [24]. Another possibility is that the aspartate substitution in the OPRM receptor resulting from 118G has different effects on its binding affinity to morphine and fentanyl due to the structural difference between the two molecules. However, this would only be plausible if 118G alters the binding affinity and subsequent downstream activities of the receptor as suggested by Bond et al [25]. Recent studies suggest that there is no difference between the two proteins in terms of binding affinity, sensitization, internalization or desensitization, but *in vitro *studies show that the there is lower expression of the receptor protein corresponding to the 118G allele [26-28].

The allele frequencies for the 118G allele for each ethnic group were similar to what we have found previously with different sets of subjects from the same three ethnic groups [15]. In the current study, the frequencies for Chinese were 0.339 compared to 0.351 in that study (p = 0.325), 0.490 compared to 0.450 for Malays (p = 0.306) and 0.441 compared to 0.474 for Indians (p = 0.450). These frequencies are similar to the other Asian populations whose reported range is 0.321 – 0.485 [21,29,30]; significantly higher than the range of 0.074 – 0.200 reported for Caucasian populations [22,30,31].

Due to the observed population difference and to rule out false association due to ethnic difference in allele frequencies, we have also analyzed the three groups separately. There was only statistically significant difference for Chinese but not the Malay and Indian subgroups. This could be due to the smaller number of subjects and the higher 118G frequencies for these two ethnic groups. Our sample size for Malays had 71% power to detect 20% difference in additive allelic effect, while our sample size for Indians had 66% power to detect 20% difference.

We have previously reported on ethnic difference in VAS scores for this study group [14]. With VAS included as a covariate, 118 A>G had the greatest contribution to morphine usage among all the variables, followed by ethnicity, but if VAS was not included as a covariate, then ethnicity would be the most significant factor, followed by OPRM. This is potentially impactful because the ethnic group that had the lowest frequency of 118G (Chinese) also used the least morphine and reported the lowest pain scores. Our finding suggests that additional genetic/environmental factors apart from 118A>G genotype could be responsible for the difference in morphine used among the three ethnic groups. As in our previous study, age and non-paying status were also predictors of higher pain scores and greater consumption of morphine for the first 24 postoperative hours. The relationship between patient's age and epidural/PCA morphine dose has been well documented [32,33]. The association of pain with age and surgical complexity was also found in a study on breast surgery [34]. The association of higher pain scores with paying status was most likely due to the surgeon's skills and experience and the duration of operation. Higher surgical complexity (usually resulting in longer duration of operation) has also been shown to correlate with higher pain scores [34].

For the OPRM promoter polymorphism, the TT group appeared to have the lowest average VAS scores. However, the number in this group was very small with only six Chinese, two Indians and no Malay having this genotype. Hence there was considerable uncertainty in the estimated medians. The potential importance of this SNP is uncertain at this point.

## Conclusion

Our results from a standardized acute post-operative pain model add to the evidence that OPRM 118 genotype influences the perception of pain and the consequent use of analgesia. We also found that ethnicity is an important factor in determining morphine requirement for post-cesarean analgesic. Identification of predictors could be important in the provision of post-surgical analgesic with morphine to provide adequate pain relief while minimizing the occurrence of side effects.

## Competing interests

The authors declare that they have no competing interests.

## Authors' contributions

ECT co-designed the study, supervised the genotyping and co-wrote the manuscript. ECPL designed the PCR-RFLP assay for the promoter polymorphism and performed all the genotyping. YYT did the statistical analyses. YL supervised the recruitment and co-designed the study and co-wrote the manuscript. HYL supervised the blood collection and DNA extraction. AS conceived the study, co-designed the study and co-wrote the manuscript.
